# A Comparative Study on the Hot Deformation Behavior of As-Cast and Twin-Roll Cast Mg-6.8Y-2.5Zn-0.4Zr Alloy

**DOI:** 10.3390/ma14133628

**Published:** 2021-06-29

**Authors:** Kristina Kittner, Madlen Ullmann, Ulrich Prahl

**Affiliations:** Institute of Metal Forming, Technische Universität Bergakademie Freiberg, Bernhard-von-Cotta-Straße 4, 09599 Freiberg, Germany; kristina.kittner@imf.tu-freiberg.de (K.K.); ulrich.prahl@imf.tu-freiberg.de (U.P.)

**Keywords:** flow curve, dynamic recrystallisation, processing map, LPSO phase, Mg-6.8Y-2.5Zn-0.4Zr, twin-roll casting, hot deformation behavior

## Abstract

The Mg-6.8Y-2.5Zn-0.4Zr (WZ73) alloy exhibits different microstructure characteristic after conventional casting compared to the twin-roll cast (TRC) state. Twin-roll casting results in a finer microstructure, where the LPSO phases are more finely distributed and less strongly connected. A transfer of the hot deformation behavior from the as-cast condition to the TRC condition is only possible to a limited extent due to the microstructural differences. Both states show differences in the recrystallization behavior during hot deformation. In the conventional cast state, dynamic recrystallization (DRX) is assumed to be delayed by the occurrence of coarse blocky LPSO phases. Main DRX mechanisms are continuous dynamic recrystallization (CDRX), particle stimulated nucleation (PSN) and twin induced dynamic recrystallization (TDRX). The deformed TRC sample showed pronounced DRX at almost all deformation conditions. Besides the TDRX and the PSN mechanism, kink induced dynamic recrystallization (KDRX) can be observed. Optimum deformation conditions for both states are temperatures from 500 °C to 520 °C, and strain rates ranging from 0.01 s^−1^ to 0.1 s^−1^ for the as-cast material as well as a strain rate of 1 s^−1^ for the TRC material.

## 1. Introduction

Within the last few years, there has been increased research on magnesium alloys with LPSO (long period stacking ordered) phases. These are known to provide alloys with a good mechanical property profile. The LPSO phases are characterized by excellent strength, unique crystal structure and good thermal stability. The good strength properties are retained even at elevated temperatures due to the high melting temperature of 545 °C. The high strength is mainly attributed to the formation of kink bands in the LPSO phase. Several studies have reported the formation of kink bands during plastic deformation for different initial states, such as as-cast or twin-roll cast. Predominantly, LPSO phases with an 18R or a 14H structure occur after conventional casting as well as after twin-roll casting (TRC) [1,2,3,4,5,6,7].

Many factors, such as temperature, strain rate and strain, are associated with the hot deformation behavior of metallic metals. For magnesium alloys containing LPSO phases, several papers are focused on the effect of such factors on workability and recrystallization behavior. Li et al. (2020) [8] investigated the hot deformation behavior of a Mg_95_._21_Zn_1_._44_Y_2_._86_Mn_0_._49_ magnesium alloy after homogenization. They have reported that at low temperatures (250 °C–300 °C), kink band formation in connection with low strain rates (0.01 s^−1^) twinning are the main deformation mechanisms. The beginning of dynamic recrystallization (DRX) at the original grain boundaries was shown at temperatures between 350 °C and 400 °C, which remains incomplete. Increasing temperatures up to 500 °C results in a complete DRX. Comparable results are presented by Zhang et al. (2018) [9] for a homogenized Mg-Gd-Y-Zn-Zr alloy. Kink deformation mainly occurs at low temperatures, where sliding systems of magnesium can hardly be activated. Fekete et al. (2020) [10] showed that twinning is still active at 300 °C during plastic deformation of a Mg_98_._5_Y_1_Zn_0_._5_ magnesium alloy. The investigations of Li et al. (2019) [11] revealed that twinning acts as an additional deformation mode even at high strain rates (0.1–1 s^−1^) during deformation of a Mg-5.6Gd-0.8Zn (wt.%) alloy. Dynamic recrystallization preferably starts at the twin boundaries (TDRX), at temperatures between 350 °C and 400 °C. Increasing temperatures lead to a change in the DRX mechanism, as new grains mainly originate at the original grain boundaries. At 500 °C the degree of DRX is considerably high. Other studies indicate that DRX starts at the primary LPSO phase [12]. It is reported that the formation of the lamellar LPSO phase can increase the critical resolved shear stress (CRSS) of the basal plane and improve the sliding of non-basal planes. Furthermore, regarding the interaction of LPSO phase and twins, Shao et al. (2020) [13] indicate that thin LPSO lamella allow the propagation of twins, while thick LPSO phases impede the twin propagation. However, the significance of the LPSO phase during hot deformation of magnesium alloys remains still unclear [10].

Based on our previous studies, this paper compares the hot deformation behavior of the Mg-6.8Y-2.5Zn-0.4Zr (WZ73) alloy in the as-cast and the twin-roll cast condition. In addition to the more economical and resource-saving production by reducing the forming and annealing effort during TRC, the quality of the cast strips can basically be improved in terms of homogeneity. The cooling rates (up to 2 106 K/s according to Allen et al. (2001) [14], i.e., about ten times faster than in slab casting), which depend on the strip thickness, have a positive effect on the microstructure and macrostructure of the strip during solidification, as well as on the precipitation state [15]. A transfer of the hot deformation behavior from the as-cast condition to the TRC condition is only possible to a limited extent, due to the microstructural differences. The purpose of this paper is to show these differences.

## 2. Materials and Methods

Cast ingots of the WZ73 alloy (Mg-6.8Y-.5Zn-0.4Zr) acquired from XI’AN YUECHEN Metal Products Co., Ltd., China, were used in this study. The chemical composition is shown in Table 1. Samples for plane strain compression tests with dimensions of 20 mm × 30 mm × 5.3 mm (length × width × height) were cut from these ingots. This condition is referred to as ‘as-cast’. The cast ingots were also used as initial material for the twin-roll casting process. TRC was conducted on the industrial pilot plant (Pechiney, France) of the Institute of Metal Forming, using a hybrid casting nozzle consisting of steel and ceramic. Important casting parameters applied were line speed of 1.625 m/min, casting temperature of 730 °C and maximum roll force of 290 kN. Detailed description of the TRC of the WZ73 strips can be found in [4]. This condition is referred to as ‘TRC’. Thickness of the TRC strip was 5.3 mm.

**Figure 1:**
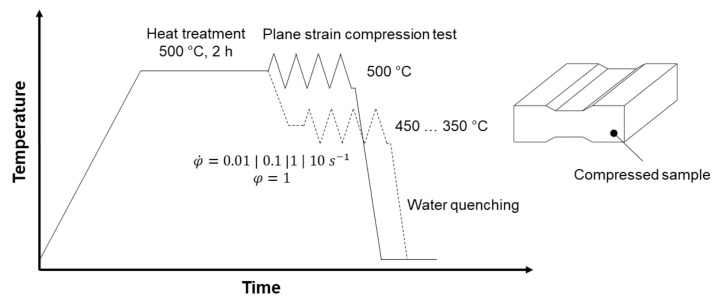
Schematic diagram of plane strain compression test.

The plane strain compression tests were performed under process-oriented conditions using a servo-hydraulic hot forming simulator. Samples were heat treated in an air circulation furnace at a temperature of 500 °C for a holding time of 2 h. Subsequent to the heat treatment, samples were compressed or cooled down (in the furnace) to the deformation temperatures between 350 °C and 450 °C and kept at these temperatures for 15 min in order to achieve a homogeneous temperature distribution over the cross section. To reduce friction between the samples surface and the tool, a graphite/oil mixture was used. Isothermal flow curves were obtained based on the recorded force-displacement data. Dissipation energy and friction were considered by the correction function according to Siebel (1932) [16], with µ = 0.12. Softening effects as a result of dissipation energy were considered numerically by using the thermodynamic temperature factor $K_{\vartheta }=\exp\left(-m_{1}\cdot \vartheta \right)$ with $m_{1}=-0.00427\;{}^{\circ}C^{-1}$ for both as-cast and TRC conditions. Continuous compression was performed at strain rates of 0.01 s^−1^, 0.1 s^−1^, 1 s^−1^ and 10 s^−1^ (Figure 1). The samples were deformed to an equivalent logarithmic strain of *φ_v_* = 1. Immediately after compression, samples were quenched into water to impede softening processes during cooling. Based on the flow curve data, processing maps are described as a function of temperature and strain rate, using the dynamic material model (DMM). The detailed approach can be found in [1,17].

From the compressed samples, longitudinal sections across the cross-section were cut and metallographically prepared for microstructural characterization. The samples were ground and then polished with an oxide polishing suspension. Etching was carried out in two steps. After brief rinsing with a 3% initial solution, the sample was etched with a picric acid solution consisting of 70 mL ethanol, 10 mL distilled water, 10 mL glacial acetic acid and 4.2 g picric acid. Light microscopic images were taken on a Keyence VHX 6000 microscope at the Institute of Metal Forming, Freiberg, Germany. Scanning electron microscopic (SEM) evaluation was performed on a ZEISS GeminiSEM 450 device at the Institute of Metal Forming, Freiberg, Germany. Different detectors were used for SEM: angular selective back-scatter (AsB), back-scatter (BSE) and secondary electron detector (SE). X-ray diffraction analysis (XRD) was performed on Empyrean Cu LFF HR at the Academic Centre for Materials and Nanotechnology, University of Science and Technology, Krakow, Poland using CuK radiation (*λ* = 1.540598 Å). Diffraction patterns were recorded within the 2*θ*-range of 30 to 60 with a step size of 0.026. For phase identification, the ICDD database was used. The diffraction peaks from Mg were identified based on ICDD 01-089-5003. However, as the existence of 18R and 14H LPSO structures cannot be unambiguously confirmed by the ICDD database, the final identification was only supported by ICDD and was evaluated based on the studies form Luo et al. (2000) [18], Onorbe et al. (2012) [19] and Wen et al. (2016) [20].

## 3. Results

### 3.1. Characterization of Initial States

**Figure 2:**
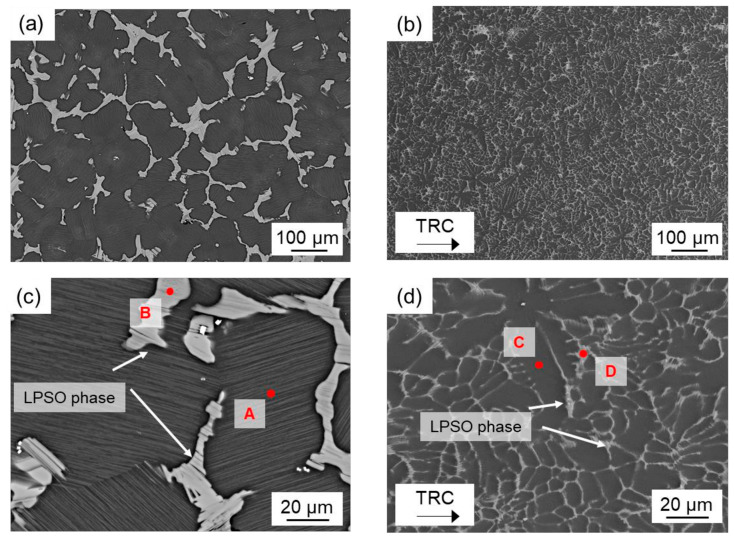
SEM images of the homogenized (500 °C, 2 h) Mg-6.8Y-2.5Zn-0.4Zr alloy: as-cast (**a**,**c**), (20 kV, AsB) and the TRC (**b**,**d**), (15 kV, SE) initial states: red points indicate EDX point measurement of the Mg matrix (A,C) and network-like LPSO structures (B,D).

**Figure 3:**
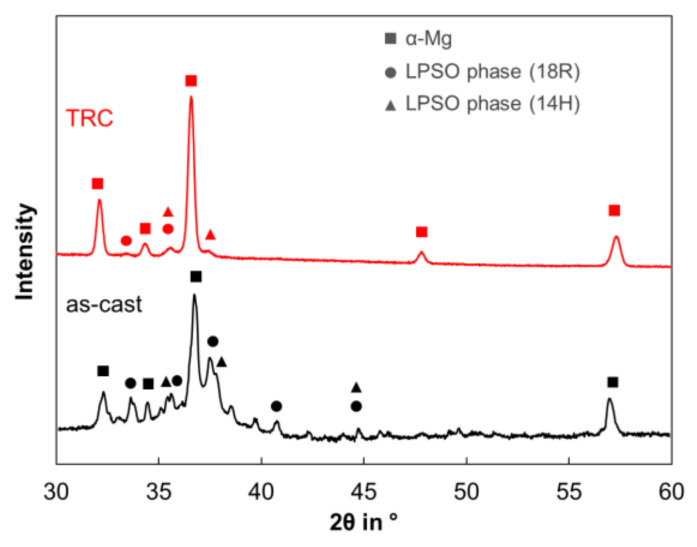
Results of the X-ray diffraction analysis (XRD) of the homogenized (500 °C, 2 h) Mg-6.8Y-2.5Zn-0.4Zr alloy after conventional and after twin-roll casting.

**Figure 4:**
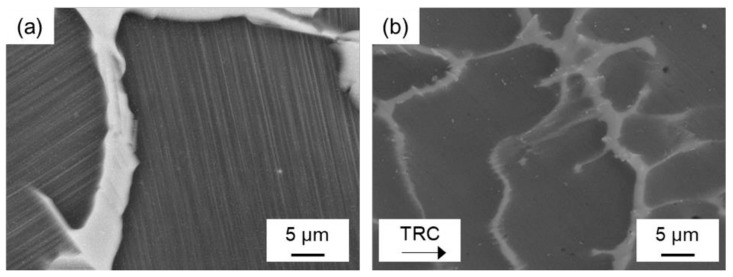
SEM images of the homogenized (500 °C, 2 h) Mg-6.8Y-2.5Zn-0.4Zr alloy after (**a**) conventional (20 kV, AsB) and after (**b**) twin-roll casting (15 kV, SE), detailed view.

Figure 2 showed the SEM-images of the microstructure of the homogenized (500 °C, 2 h) Mg-6.8Y-2.5Zn-0.4Zr alloy after conventional and after twin-roll casting. The microstructure of both conditions mainly consists of the α-magnesium matrix (dark grey) and network-like LPSO phases (light grey) located along the grain boundaries. Twin-roll casting leads to a finer microstructure compared to conventional casting. Thickness of the blocky LPSO phases within the network varies from 2 µm to 5 µm in the TRC (Figure 2d), while it rises up to 20 µm in the as-cast state (Figure 2c). According to the results of the X-ray diffraction analysis (Figure 3) and EDX analysis (Table 2), LPSO phases could be assigned to an 18R or 14H structure. The exact distinction of both kinds of LPSO phases requires transmission electron microscopy. Based on the literature, the results are consistent with those of other research groups investigating magnesium alloys consisting of LPSO phases [21,22,23]. A detailed view into the microstructure (Figure 4) reveals another substantial difference between both initial states. After conventional casting, thin lamellar LPSO phases arise within the α-magnesium grains (Figure 4a). It is reported that those lamellae are enriched with Y and Zn [24], and can be assigned to the 14H structure [6,25]. It is assumed that the 14H LPSO lamellae can grow from stacking faults (SF) which precipitated from a supersaturated Mg matrix. During twin-roll casting and subsequent annealing, the formation of lamellar LPSO phases cannot be observed (Figure 4b). It is therefore assumed that the high cooling rate during TRC and the characteristic solidification conditions have a major impact on the precipitation kinetics of the LPSO phases. The amount of Y and Zn within the magnesium matrix after conventional casting is 3.6 wt.% and 1.4 wt.%, respectively, while it is 2.2 wt.% and 0.7 wt.% in the TRC state. Higher amounts of solute atoms in the magnesium matrix after TRC can be achieved by heat treatments at high temperatures above 500 °C and long holding times above 6 h. Previous studies showed that the amount of Y and Zn increased to 3.8 wt.% Y and 1.4 wt.% Zn after homogenization at 525 °C for 6 h. Followed by a low cooling rate, thin lamellar LPSO phases precipitate within the magnesium matrix [4].

### 3.2. Hot Deformation Behavior

#### 3.2.1. Flow Stress Behavior

**Figure 5:**
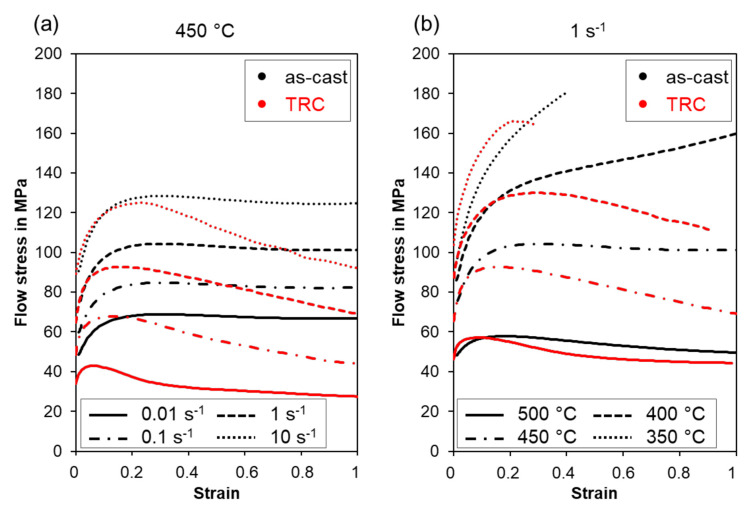
Flow stress-strain curves of the homogenized Mg-6.8Y-2.5Zn-0.4Zr alloy after conventional and twin-roll casting, deformed at (**a**) a temperature of 450 °C and different strain rates (0.01, 0.1, 1, 10 s^−1^), and at (**b**) a strain rate of 1 s^−1^ and different temperatures (350, 400, 450, 500 °C).

Figure 5 shows the flow stress–strain curves of the homogenized Mg-6.8Y-2.5Zn-0.4Zr after conventional and twin-roll casting deformed under different temperatures (350 °C, 400 °C, 450 °C, 500 °C) and strain rates (0.01, 0.1, 1, 10 s^−1^) during hot compression. At a deformation temperature of 450 °C (Figure 5a) the flow stress–strain curves offer similar characteristics and the curves can be separated in three distinct regions. According to the occurring deformation mechanisms these regions correspond to hardening, transition and softening [26]. At the beginning of the flow, curve strain hardening is dominant and depends on the dislocation structure originating from plastic deformation. The increasing flow stress results from the dislocation storage, for example at the interface of α-Mg and LPSO phase. At low strain rates (<1 s^−1^), the beginning of the plastic flow is accompanied by higher flow stresses in the as-cast condition, while at 10 s^−1^ the TRC and as-cast condition exhibit the same flow stress at the beginning of the plastic flow. Maximum flow stress in the as-cast state is higher compared to the TRC state. It is assumed that at the large-scale LPSO phases in the as-cast state the dislocation density is higher because dislocations can hardly overcome or circumvent those LPSO phases compared to the finer phases after TRC. Therefore, in the as-cast condition, the flow curve exhibits higher flow stresses at 450 °C.

**Figure 6:**
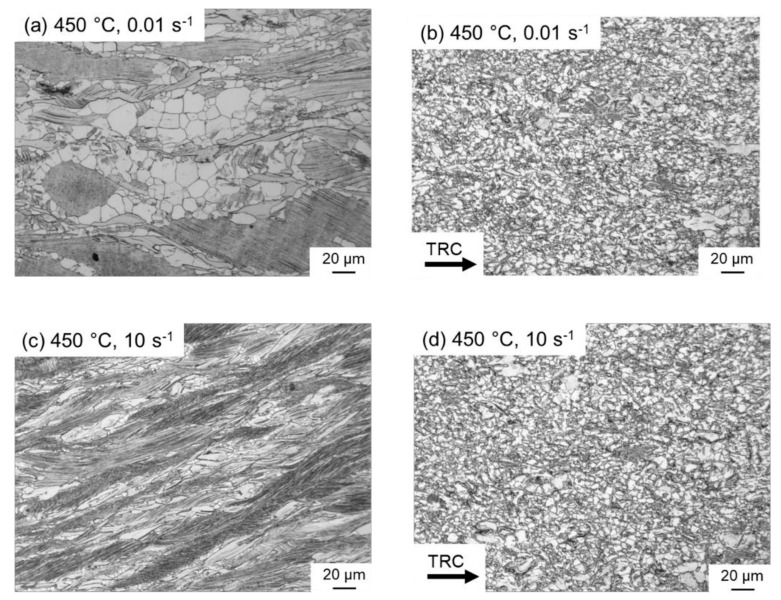
Optical micrographs of the deformed samples of the Mg-6.8Y-2.5Zn-0.4Zr alloy after conventional (**a**,**c**) and after twin-roll casting (**b**,**d**) at 450 °C and strain rates of 0.01 s^−1^ (**a**,**b**) and 10 s^−1^ (**c**,**d**).

After reaching the peak stress, the flow decreases or remains at a certain level because of softening processes, which take place when plastic deformation is maintained. During softening, dislocation movement is enabled by cross-slip and climbing processes, which result in the annihilation of dislocations and in a decreasing dislocation density. Both lead to a decrease of the flow stress. The flow curves of the as-cast condition reveal only a slight decrease of the flow stress, and then it is in a quasi-steady state, where hardening and softening counteract each other. It is assumed that at 450 °C dynamic recovery is the dominant softening mechanism. Only a small amount of dynamic recrystallization can be observed, indicating that the DRX remains incomplete even at high strains and low strain rates (Figure 6a,c). After twin-roll casting, the softening behavior deviates from the above. Once the maximum flow stress is exceeded, the flow curve drops significantly, which could be attributed to dynamic recrystallization (Figure 6b,d). The micrographs show an almost completely recrystallized structure at both high (10 s^−1^) and low (0.01 s^−1^) strain rates.

Decreasing forming temperatures result in increasing flow stress, which is shown in a temperature range from 350 °C to 500 °C and a strain rate of 1 s^−1^ in Figure 5b. The Mg-6.8Y-2.5Zn-0.4Zr alloy in the as-cast state exhibits an enhanced hardening behavior during plastic deformation at 350 °C and 400 °C. Softening during compression could not be obtained, and the samples deformed at 350 °C failed at a strain of 0.4. The TRC material revealed that softening occurred after reaching the peak stress even at low deformation temperatures (350 °C–400 °C). However, at 350 °C sample failed at a strain of 0.3, because enhanced hardening in the early stage of deformation results in the formation of microcracks, which provided the starting point for the material failure. From temperatures up to 400 °C, the flow curves show a characteristic progression of hardening and softening, with DRX predominating. Differences in temperature-dependent hardening and softening behavior between as-cast and TRC conditions almost disappear at 500 °C. At 500 °C, the flow curve of the conventional cast material also shows a softening behavior typical of dynamic recrystallization. For magnesium alloys containing LPSO phases, several research groups investigated the hot deformation behavior and presented comparable results. Li et al. (2020) [8] showed flow stress-strain curves for a Mg_95_._21_Zn_1_._44_Y_2_._86_Mn_0_._49_ magnesium alloy, which are typical for DRX during hot compression. Main deformation mechanisms were kink deformation and twinning, while dynamic recrystallization occurred along the original grain boundaries and in the vicinity of the LPSO phases. Flow curves of the Mg-5.6GD-0.8Zn (wt.%) alloy reveal DRX characteristics expect hot deformation at 350 °C and 0.1 s^−1^ to 1 s^−1^. At higher strain rates, twinning is an additional deformation mode and DRX takes place at the twin boundaries [11]. Lv et al. (2014) [27] investigated the hot deformation behavior of a Mg-2.0Zn-0.3Zr-5.8Y (wt.%) alloy in the temperature range from 300 °C to 500 °C and strain rates from 0.001 s^−1^ to 1 s^−1^. The material showed a characteristic behavior of strain hardening and flow softening at low temperatures and high strain rates, while the flow stress decreases to a steady state at high temperatures and low strain rates.

#### 3.2.2. Constitutive Equation

The activation energy is an important material parameter for determining the critical conditions for initiating dynamic recrystallization. In determining the model coefficients, it is assumed that the processes taking place during hot deformation are diffusion-controlled and thus strain rate- and temperature-dependent. The calculated flow curves form the basis for determining the dynamic recrystallization processes. From the flow stress maxima determined, the Zener–Hollomon parameter Z is established, considering the model coefficients A, α and n as well as the activation energy Q, using Equation (1) according to Sellars (1966) [28,29]:

(1) $$Z=\;\dot{\varepsilon }\;\exp\left\{\frac{Q}{R\cdot T}\right\}=A{\left[\sin\left(\alpha \sigma \right)\right]}^{n}$$

where $\dot{\varepsilon }$ is the strain rate (s^−1^), σ is the flow stress (MPa), A and α are material constants, n is the stress exponent, R is the universal gas constant (8.314 J·K^−1^mol^−1^), and T is the absolute deformation temperature (K). Values for A, α and n for the as-cast and the TRC states can be found in Table 3. The parameters of the equation were determined using the mean values from the increases in the graphs, which represented the linear relationship of the flow stress to the comparative strain rate and temperature.

**Figure 7:**
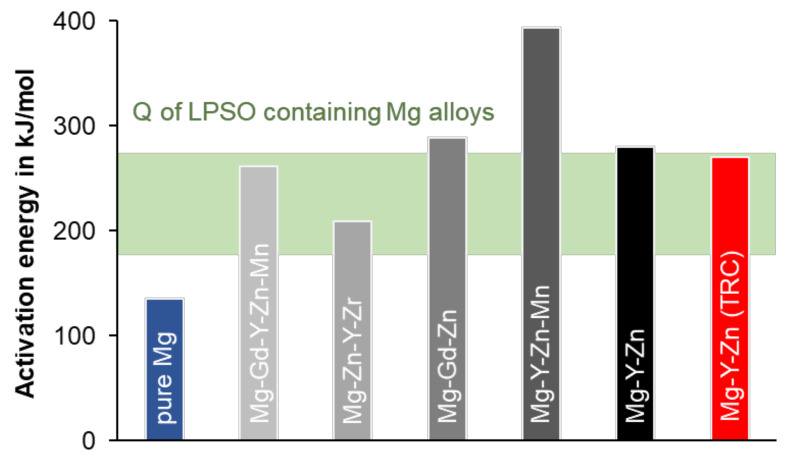
Activation energy Q of LPSO containing magnesium alloys compared to pure Mg [8,11,30,31,33,34].

The activation energy describes the difficulty of initiating deformation during hot working and is simultaneously affected by dynamic precipitation formation, pinning effects of dislocations, and the occurrence of secondary phases. The activation energy for dynamic recrystallization of LPSO containing magnesium alloys derived by different scientists is very different and varies between 182 kJ/mol and 276 kJ/mol, according to Gonzales et al. (2016) [30]. These values are significantly higher than the activation energy for self-diffusion in magnesium (135 kJ/mol [31]). Figure 7 shows the activation energies for various Mg alloys with LPSO phases. Only Mg-Y-Zn-Mn [8] and Mg-Gd-Zn [11] offer higher values with 393 kJ/mol and 289 kJ/mol, respectively. The activation energy for the Mg-6.8Y-2.5Zn-0.4Zr alloy investigated in this work is 279 kJ/mol for as-cast and 270 kJ/mol for the TRC conditions, and is thus in the upper range. Possible origins of the high Q-values for the Mg-Y-Zn alloys lie in the presence of the LPSO phase, which can occupy a proportion of up to 20% [4], depending on the alloy composition. The LPSO phases impede dislocation movement during deformation and cause self-diffusion within the crystal lattice to be suppressed. The influence of the proportion of alloying elements becomes clear when comparing the activation energy with twin-roll cast AZ31. Investigations by [32] show a value of Q = 178 kJ/mol.

#### 3.2.3. Deformation and Recrystallization Behavior

**Figure 8:**
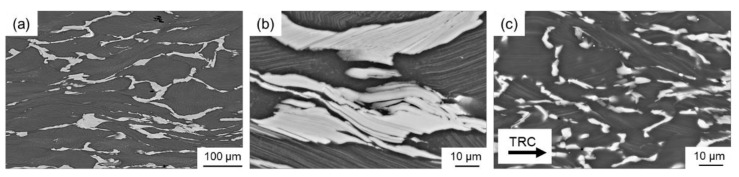
SEM images (15 kV, SE) of the deformed samples (**a**) as-cast condition, overview, (**b**) as-cast condition, detail and (**c**) TRC condition, detail.

**Figure 9:**
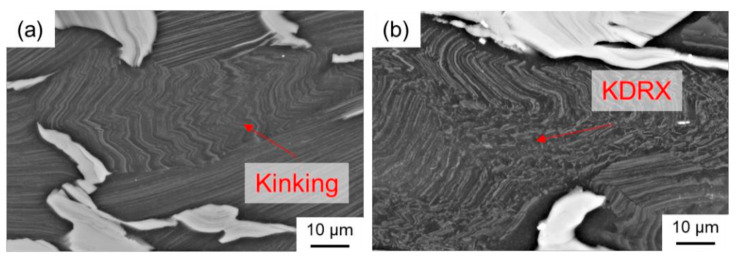
SEM images (20 kV, BSD) of deformed samples of the as-cast condition: (**a**) kinking at 350 °C and (**b**) kink-aided dynamic recrystallization at 450 °C.

**Figure 10:**
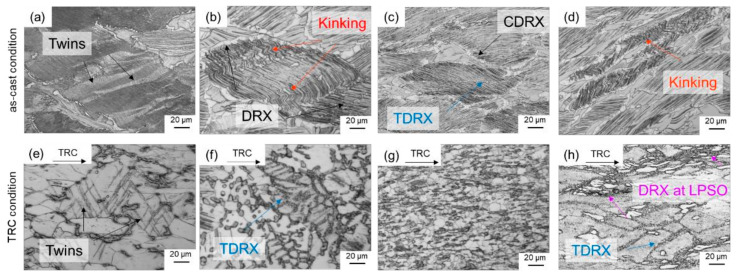
Optical micrographs of the deformed samples after conventional casting (**a**–**d**) and TRC (**e**–**h**) showing different deformation and recrystallization mechanisms.

The LPSO phases occur in different forms, block-like, connected to networks or isolated as well as in lamellar form. Due to their high thermal stability, a high density of LPSO phases remains in the microstructure even after hot deformation at temperatures between 350 °C and 500 °C. Figure 8 shows SEM images of the as-cast and the TRC condition after hot deformation, revealing differences in the nature of the LPSO phases that significantly affect recrystallization behavior. In the deformed sample after conventional casting (Figure 8a), the LPSO phases appear predominantly as coarse block-like structures that are interconnected in a network. These consecutive LPSO phases disrupt the boundary migration of the DRX nuclei and have a hindering effect on the dynamic recrystallization. Simultaneously, lamellar LPSO phases occur within the magnesium matrix grains. As a result of hot deformation, the lamellae were kinked (Figure 9) and exhibit zigzag-like morphologies (Figure 10b,d). The initiation and arrangement of basal pairs of dislocations that align perpendicular to the slip plane are suggested to be the origin for the formation of kink boundaries [35]. The stacking sequence of the lamellar 14 H LPSO phase is suggested to restrict dynamic recrystallization and the occurrence of twinning within the matrix grains [36]. Therefore, in the as-cast state, fine DRXed grains are mainly formed at the initial grain boundaries as a result of continuous dynamic recrystallization (CDRX), and in the vicinity of the block-shaped LPSO phase via particle stimulated nucleation (PSN). However, PSN is less pronounced due to the suppressive effect caused by the nature of the blocky LPSO phases. Consequently, a bimodal microstructure consisting of coarse deformed original grains as well as fine dynamic recrystallized grains develops (Figure 10b,c). Contrary to the assumption of Hagihara et al. (2010) [36], twins can be observed within the magnesium matrix despite the presence of the lamellar LPSO phase (Figure 10a). These also serve as starting points for the dynamic recrystallization (TDRX, Figure 10c). Based on these results, DRX is assumed to be delayed and the main recrystallization mechanisms of the as-cast conditions are CDRX, PSN and TDRX. Kink-aided dynamic recrystallization (KDRX), like it was reported by Chen et al. (2020) [37] can hardly be detected. They showed that KDRX mainly occurs, where the LPSO phase is fairly fragmented. In this work, at low temperatures (350 °C) LPSO lamellae are kinked but not fragmented (Figure 9a). As can be seen from Figure 9a, kinking only takes place when lamellae are aligned parallel to compression direction. Fragmentation of lamellar LPSO phases can only be observed at temperatures above 450 °C (Figure 9b), and KDRX can be found. However, only a few grains exhibit lamellae in the compression direction and, at the same time, fragmentation of the lamellae, so the number of grains in which KDRX takes place is limited.

**Figure 11:**
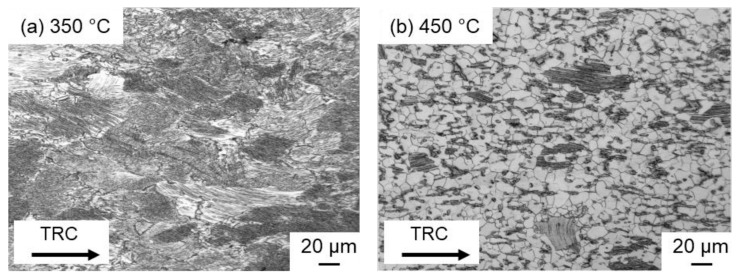
Optical micrographs of the deformed samples after twin-roll casting compressed at different temperatures (**a**) 350 °C and (**b**) 450 °C and a strain rate of 10 s^−1^.

In the TRC initial state, the block-like LPSO phase is more finely distributed and, above all, less strongly connected. The deformed specimen shows that the blocky LPSO phase is aligned in the flow direction of the material. At the same time, previous investigations [4] show that, depending on the deformation temperature, lamellar LPSO phases are precipitated within the Mg matrix during cooling from the heat treatment (Figure 11). It can be clearly seen that at 350 °C almost the entire Mg matrix is equipped with lamellae (Figure 11a), while at 450 °C only a few grains have lamellar LPSO phases (Figure 11b). This results in different recrystallization mechanisms depending on the deformation temperature.

**Figure 12:**
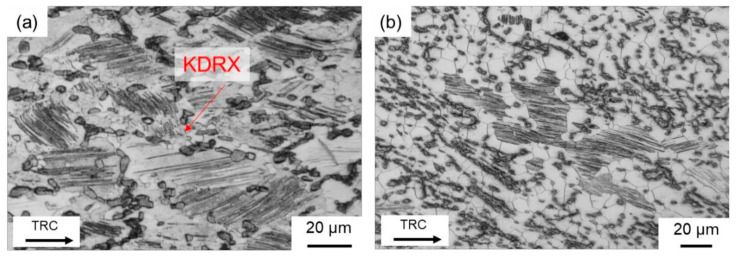
Optical micrographs of the deformed samples after twin-roll casting: (**a**) KDRX at fragmented lamellar LPSO phases and (**b**) grains with lamellar LPSO phases remain, while the surrounding microstructure is almost completely recrystallized.

When deformation takes place at 350 °C, hardly any DRX occurs. However, it was also found in previous investigations [38] that twin and lamellar LPSO phases occur simultaneously. As the deformation temperature increases, the number of grains exhibiting lamellar LPSO phases decreases. Twins continue to occur (Figure 10e). Twin boundaries serve as starting points for dynamic recrystallization (TDRX) during hot deformation (Figure 10f,h). In the TRC initial state, DRX at the blocky LPSO phases plays a more important role. Their fine distribution and less network-like nature favor DRX by PSN (Figure 10h). Comparable results are also reported by Onorbe et al. (2012) [19]. Thus, a uniformly recrystallized microstructure can be achieved (Figure 10g). In deformed specimens exhibiting lamellar LPSO phases and kinking, DRX can be observed sporadically at the kink boundaries. Comparable to the studies of Chen et al. (2020) [37], KDRX occurs when the lamellae are fragmented (Figure 12a). However, this phenomenon occurs rather rarely. Figure 12b shows that LPSO lamellae without kinking have a rather unfavorable effect on the DRX. The grains with lamellae remain in the almost completely recrystallized microstructure.

#### 3.2.4. Processing Maps

**Figure 13:**
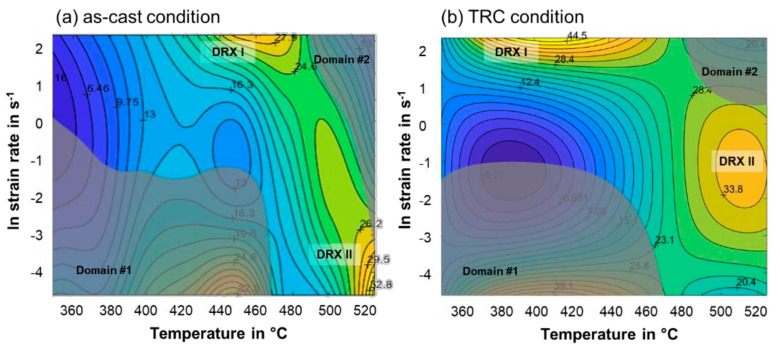
Processing maps of the (**a**) as-cast condition and (**b**) TRC condition in the temperature range from 350 °C to 525 °C at a strain of 0.4 [1,38].

The DMM processing maps at a moderate strain of 0.4 are shown in Figure 13a for the as-cast, and in Figure 13b, for the TRC condition. Processing maps are based on an overlap of the power dissipation maps with the instability one according to [39]. The yellow regions possess high power dissipation efficiency of about 27% to 30% for the as-cast and 34% to 44% for the TRC condition. Both conditions offer a high efficiency at high strain rates (5 s^−1^) in a temperature range of 400 °C to 440 °C for TRC condition, while for as-cast conditions higher temperatures of 450 °C to 470 °C are required for higher power dissipation efficiency. At higher temperatures (500 °C to 520 °C) moderate strain rates of 1 s^−1^ (TRC condition) and low strain rates of 0.1 s^−1^ to 0.01 s^−1^ (as-cast condition) are preferable for hot deformation. Under these conditions, dynamic recrystallisation is assumed to occur preferentially during hot deformation.

**Figure 14:**
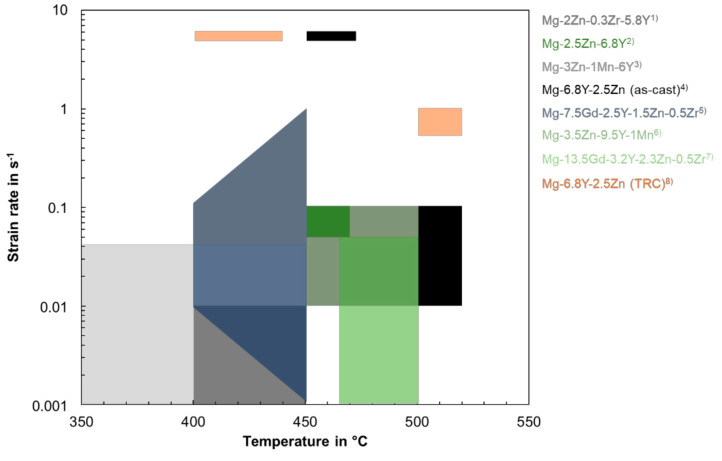
Process windows for several LPSO containing magnesium alloys based on processing maps: (1) [27] (2) [40] (3) [41] (4) [1] (5) [42] (6) [8] (7) [9] (8) [38].

Literature data on suitable hot deformation conditions for comparable alloys containing LPSO phases are presented in Figure 14. High efficiency regions are located at temperatures below 500 °C and strain rates lower than 1 s^−1^. The conventionally cast and TRC Mg-6.8Y-2.5Zn-0.4Zr alloy both show flow instabilities in this parameter range. A second instability domain occurs at high temperatures (>500 °C). For the TRC condition unstable flow is restricted to high strain rates (10 s^−1^), while it is broadened for the as-cast condition to lower strain rates (0.01 s^−1^). In these zones, where the corresponding power dissipation coefficient is comparatively low, microcracks nucleate and propagate along the interface of the grain boundaries and the network-like LPSO phase. At low temperature and/or high strain rates, a large quantity of dislocations could pile-up at the interface of the magnesium matrix and the LPSO phase. The instability of adiabatic shear bands or local plastic flow tends to occur rather than dynamic recrystallization. Consequently, these instabilities result in the formation of microcracks [1,38]. Optimum deformation conditions are temperatures from 500 °C to 520 °C at strain rates ranging from 0.01 s^−1^ to 0.1 s^−1^ for the as-cast material and a strain rate of 1 s^−1^ for the TRC material.

## 4. Conclusions

This paper compares the hot deformation behavior of the Mg-6.8Y-2.5Zn-0.4Zr (WZ73) alloy in the as-cast and the twin-roll cast condition. The main findings can be summarized as follows:

- The microstructure of both conditions mainly consists of the α-magnesium matrix and network-like LPSO phases located along the grain boundaries. LPSO phases could be assigned to 18R or 14H structures. Twin-roll casting leads to a finer microstructure compared to conventional casting. After conventional casting, thin lamellar LPSO phases arise within the α-magnesium grains, which can be assigned to the 14H structure. In the TRC condition, lamellar LPSO phases are not observed.
- During hot deformation of the conventionally cast material, it is assumed that at 450 °C dynamic recovery is the dominant softening mechanism. Only a small amount of dynamic recrystallization can be observed, indicating that the DRX remains incomplete even at high strains and low strain rates. The TRC samples showed softening via dynamic recrystallization, where an almost completely recrystallized structure developed at both high (10 s^−1^) and low (0.01 s^−1^) strain rates.
- At higher temperatures (500 °C), DRX occurs in both conditions. The as-cast state exhibits a high amount of network-like and lamellar LPSO phases, which are assumed to be responsible for a delayed DRX. The main recrystallization mechanisms of the as-cast condition are CDRX, PSN and TDRX. Kink-aided dynamic recrystallization can hardly be detected. In the TRC initial state, the block-like LPSO phase is more finely distributed and, above all, less strongly connected. Lamellar LPSO phases are precipitated within the Mg matrix during cooling and different DRX mechanism take place depending on deformation temperature. The fine distribution of the LPSO phase and less network-like nature favor DRX by PSN. Besides this, TDRX and KDRX can be observed.
- Optimum deformation conditions are temperatures from 500 °C to 520 °C and strain rates ranging from 0.01 s^−1^ to 0.1 s^−1^ for the as-cast material as well as a strain rate of 1 s^−1^ for the TRC material.

## Figures and Tables

**Table 1:** Chemical composition of Mg-6.8Y-2.5Zn-0.4Zr (wt.%) alloy as determined by means of optical emission spectrometry (OES, Spectro/Ametek, Germany).

Y	Zn	Zr	Si	Fe	Cu	Ni	Others	Mg
6.8	2.5	0.4	0.01	0.005	0.001	0.001	0.01	Balance

**Table 2:** Chemical composition of microstructural constituents from the as-cast and the TRC condition in Figure 2 (A, C—magnesium matrix, B, D—network-like LPSO structures) determined by means of energy dispersive X-ray spectroscopy (EDX)-analysis (wt.%).

Name		Mg	Y	Zn
A	matrix as-cast	95.0	3.6	1.4
B	LPSO as-cast	73.2	17.2	9.6
C	matrix TRC	97.1	2.2	0.7
D	LPSO TRC	74.2	15.6	10.2

**Table 3:** Model coefficients for determination of Z according to Equation (1).

Model Coefficient	As-Cast State	TRC State
A in s^−1^	5.33817 × 10^19^	2.61662 × 10^19^
α in MPa^−1^	0.010677	0.010849
n	6.6051	7.4871
